# Deaths, late deaths, and role of infecting dose in Ebola virus disease in Sierra Leone: retrospective cohort study

**DOI:** 10.1136/bmj.i2403

**Published:** 2016-05-18

**Authors:** Hilary Bower, Elizabeth Smout, Mohamed S Bangura, Osman Kamara, Cecilia Turay, Sembia Johnson, Shefali Oza, Francesco Checchi, Judith R Glynn

**Affiliations:** 1 Department of Infectious Disease Epidemiology, London School of Hygiene and Tropical Medicine, London, UK; 2 Save the Children, Freetown, Sierra Leone; 3 Humanitarian Department, Save the Children, London, UK; 4 London School of Hygiene and Tropical Medicine, London WC1E 7HT, UK

## Abstract

**Objectives** To assess the frequency of fatal recrudescence from Ebola virus disease after discharge from treatment centres, and explore the influence of infecting dose on case fatality rates.

**Design** Retrospective cohort study.

**Setting** Western Area, Sierra Leone.

**Participants** 151 survivors treated for Ebola virus disease at the Kerry Town treatment centre and discharged. Survivors were followed up for a vital status check at four to nine months after discharge, and again at six to 13 months after discharge. Verbal autopsies were conducted for four survivors who had died since discharge (that is, late deaths). Survivors still living in Western Area were interviewed together with their household members. Exposure level to Ebola virus disease was ascertained as a proxy of infecting dose, including for those who died.

**Main outcome measures** Risks and causes of late death; case fatality rates; odds ratios of death from Ebola virus disease by age, sex, exposure level, date, occupation, and household risk factors.

**Results** Follow-up information was obtained on all 151 survivors of Ebola virus disease, a mean of 10 months after discharge. Four deaths occurred after discharge, all within six weeks: two probably due to late complications, one to prior tuberculosis, and only one after apparent full recovery, giving a maximum estimate of recrudescence leading to death of 0.7%. In these households, 395 people were reported to have had Ebola virus disease, of whom 227 died. A further 53 people fulfilled the case definition for probable disease, of whom 11 died. Therefore, the case fatality rate was 57.5% (227/395) for reported Ebola virus disease, or 53.1% (238/448) including probable disease. Case fatality rates were higher in children aged under 2 years and adults older than 30 years, in larger households, and in infections occurring earlier in the epidemic in Sierra Leone. There was no consistent trend of case fatality rate with exposure level, although increasing exposure increased the risk of Ebola virus disease.

**Conclusions** In this study of survivors in Western Area, Sierra Leone, late recrudescence of severe Ebola virus disease appears to be rare. There was no evidence for an effect of infecting dose (as measured by exposure level) on the severity of disease.

## Introduction

Understanding who dies from Ebola virus disease is crucial for determining the effect of interventions and planning the public health response. The case fatality rate for the disease is high but estimates have varied between outbreaks and in reports describing the west African outbreak. In previous outbreaks, case fatality rates have been between 34% and 88%, with generally lower rates for the *Sudan* and *Bundibugyo ebolavirus* species than for *Zaire ebolavirus*.^1^ ^2^ ^3^ In the west African outbreak of *Zaire ebolavirus*, the case fatality rate based on the notification data for certain and probable cases was 65%, slightly lower in Guinea and higher in Liberia than in Sierra Leone, but some cases may not have been notified.^4^ Estimates from west African treatment centres have ranged from 31%^5^ to more than 70%,^6^ but patients who die or recover without reaching the centres are not included and variation reflects admission policies and delays, and patient mix as well as care. Community level data should give the best estimates but there are few such studies,^7^ ^8^ and to ensure unbiased estimates they would need to include any unreported mild cases and assessment of any unreported deaths.

Late deaths due to Ebola complications or recrudescence of the virus would also be excluded from estimated case fatality rates. The recrudescence of Ebola virus disease in a nurse in the United Kingdom, nine months after the original episode, raised the possibility that similar events are occurring but are being missed in west Africa, where they might be fatal.^9^ In Liberia, a nine year old child was readmitted with meningoencephalitis and a polymerase chain reaction (PCR) test that was positive for Ebola virus one day after discharge with negative blood tests.^9^ Recrudescences are important not only for the individuals but also as a possible source of further outbreaks. The frequency of severe recrudescence leading to late deaths is not known, although it has been noted that the virus can persist in protected body sites for at least nine months.^10^ Studies of post-Ebola sequelae have so far concentrated on survivors attending clinics,^11^ ^12^ or have not managed to contact all survivors^13^: unless intensive follow-up is conducted, deaths could be missed.

Several studies have looked at risk factors for death from Ebola virus disease. There is a clear association with age in the larger studies, with the lowest case fatality rates in children aged over 4 years and high rates in children aged under 2 years and older adults, and little difference by sex.^2^ ^4^ Some studies have found case fatality rates decreased over the course of an outbreak,^2^ ^8^ perhaps reflecting improved care. Survival in those in treatment centres is better than overall survival,^8^ ^14^ but whether this reflects the treatment or the selection of those surviving long enough to get to centres is not clear. Among patients in the treatment centres, the key clinical predictor of mortality is the estimated viral load on arrival.^14^ ^15^ ^16^

In the first known Ebola outbreak, in Yambuku, Democratic Republic of Congo, case fatality rates were higher in patients who acquired the infection following injection (100%, 85/85) than by contact (80%, 119/149, P<0.001).^17^ This comparison was not adjusted for other factors but the age distribution of patients infected by injection and by contact was similar.^17^ The association between route of infection and mortality, and the strong correlation between viral load on admission to treatment centres and mortality, suggest an effect of infecting dose on severity of disease—as found, for example, for measles.^18^ A dose effect could also explain the lower case fatality rate in older children, if they are less exposed. The effect of infectious dose on severity of disease has not been investigated previously for Ebola virus disease.

In this retrospective cohort study, we assess risk factors for death from Ebola virus disease, including level of exposure to individuals with Ebola virus disease and their body fluids as a proxy of infecting dose. We also assess the frequency of late deaths in those patients discharged as survivors.

## Methods

As part of a retrospective cohort study of transmission patterns, all survivors (or their parents or guardians) who were discharged from the Kerry Town Ebola treatment centre between November 2014 and March 2015 were sought and asked to attend an interview, together with anyone who was living with them at the time that anyone in their household had Ebola virus disease. All the people living in the household at that time were enumerated, and their age, sex, and whether they had had or died from Ebola virus disease was recorded. For those household members who were not said to have had Ebola virus disease, we asked about symptoms at that time. For those discharged as survivors following negative PCR tests for the virus, but who were subsequently found to have died, a verbal autopsy with family members was conducted by a physician, and we examined medical notes and sought information from the treating physicians, where available. The verbal autopsies used a modified version of the World Health Organization’s 2014 verbal autopsy instrument. Households were sought for interview between June and September 2015, and again between December 2015 and January 2016 to confirm vital status and conduct verbal autopsies. Individual, written informed consent was sought before interviews, with consent from parents or guardians for those aged under 18 years.

To estimate the level of exposure to Ebola virus, we asked household members to describe in their own words what happened when the Ebola infection struck. For each person with Ebola virus disease, we asked what symptoms they had had, who had taken care of them, who helped them with different activities, who shared a bed with them, among other details. We also asked about any external contacts. The aim was to identify the extent of contact with possibly infective body fluids. Using an eight level scale, we assigned the maximum contact level for each person in the household. We predefined this scale on the basis of the available literature and in discussion with frontline health workers working with individuals with Ebola virus disease. Exposure, from the highest to lowest levels, was defined as follows:

- Direct contact with, or touching, the body of a person who died of Ebola virus disease
- Direct contact with the body fluids of a patient who has Ebola virus disease with wet symptoms (that is, diarrhoea, vomiting, or bleeding)
- Direct contact with a patient with wet symptoms (eg, sharing a bed, providing care, embracing, carrying)
- Direct contact with a patient with dry symptoms (that is, without wet symptoms)
- Indirect contact with a patient with wet symptoms (eg, washing their clothes)
- Indirect contact with a patient with dry symptoms
- Minimal contact (eg, shared meals)
- No known contact

We defined individuals with Ebola virus disease as those already known as survivors from the Kerry Town treatment centre, reported by their families to be survivors from other treatment centres, or reported to have died of the disease. In addition, we included individuals (living or dead) not reported as having had Ebola virus disease but who had symptoms fitting the Sierra Leone case definition of probable disease,^19^ unless they had had a negative PCR test at the time. We assessed the effect of including people with probable disease in a sensitivity analysis. Recrudescence of Ebola virus disease was defined as illness or death that could not be attributed to a non-Ebola related cause after a period of full recovery from confirmed Ebola virus disease.

Analyses of risk factors for death used multiple logistic regression, adjusting for clustering by household using random effects. In addition to age, sex, and exposure level, we assessed other risk factors for associations with the outcome of Ebola virus disease. These factors included first or subsequent case in the household, date of Ebola virus infection in household, position in household (head or member), occupation, number of people in the household, and household living conditions (as a score based on measures of crowding and sanitation (access to water, soap, and latrine)). Age, sex, and exposure level were kept in the multivariable model a priori. We added other variables one by one and retained them in the model if they were associated with mortality. We repeated the analyses excluding those cases and deaths classified as probable Ebola virus disease but not reported as Ebola virus disease by the family. We used Stata 14 for analysis.

### Patient involvement

Two survivors of Ebola virus disease were involved in the development of the questionnaire and the implementation of the study and were asked to advise on interpretation and writing up of results. There are no plans to disseminate the results of the research directly to the study participants or the relevant patient community, but we will disseminate results to the Ministry of Health and Sanitation and the Ministry of Social Welfare, Gender and Children’s Affairs in Sierra Leone, who have responsibility for Ebola survivors.

## Results

### Late deaths

We obtained follow-up information on all 151 survivors who had been discharged from the Kerry Town Ebola treatment centre with negative blood tests, either from direct contact, or from families or other informants. Four of these survivors had died. The others were known to be alive for a mean of 10 months (range six to 13) after discharge. Details of the four late deaths are as follows:

- Patient A: a 25 year old woman who died 15 days after discharge. During admission, she showed signs of hepatitis. Her liver function tests had greatly improved before discharge but her amylase level was very high. At discharge, her family reported that she was unable to walk but could crawl. She “felt fine” for two days but then developed abdominal swelling, diarrhoea, and swelling of the legs and face, and she looked pale and jaundiced. A postmortem swab by the burial team was found to be negative for Ebola virus by PCR.
- Patient B: a 32 year old woman who died one day after discharge. She was very confused on admission, then improved but continued to act strangely. She had high blood pressure on some days but not consistently. Her platelet count was normal. At discharge, she was unable to walk. The following day, she had a sudden severe headache and was unable to talk or use her limbs. She died that evening. A postmortem swab by the burial team was found to be negative for Ebola virus by PCR.
- Patient C: a 17 year old boy who died five weeks after discharge. His health was reported to have returned to normal after discharge. He then developed weight loss, night sweats, and a productive cough that started after discharge. One week before death, he had pain and difficulty swallowing solids but no other specific symptoms. He died in his sleep. A postmortem swab by the burial team was found to be negative for Ebola virus by PCR.
- Patient D: a 6 year old boy who died one week after discharge. He had had a cough for several months before having Ebola virus disease. On recovery, he remained short of breath with a productive cough and fluctuating pyrexia that did not respond to antibiotics. He was transferred to a paediatric hospital for investigation of possible tuberculosis. A chest radiograph was compatible with miliary tuberculosis. A postmortem PCR test was borderline positive for Ebola virus.

### Household members

Of the 151 Kerry Town survivors sought for interview in June to September 2015, eight were living outside Western Area. We did not seek to interview households of survivors known to have died after discharge, except for patient A (because there was another survivor in the household). One survivor refused to take part and 16 were unavailable or not contactable at that time. Therefore, the remaining 123 survivors (including patient A) were included in the study. 

The 123 survivors lived in 94 households with 816 household members. We excluded four household members whose cause of death was unclear (fig 1). Overall, 395 people were reported to have had Ebola virus disease in these households (including patients treated at other facilities), of whom 227 died (excluding patient A). A further 53 people fulfilled the case definition for probable Ebola virus disease, of whom 11 died. Therefore, the case fatality rate was 57.5% (227/395) for reported Ebola virus disease, or 53.1% (238/448) including probable disease.

**Figure f1:**
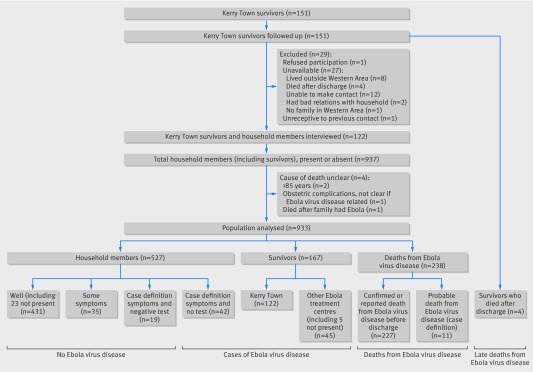
**Fig 1** Flow diagram showing study composition of participants, from households of survivors of Ebola virus disease

Figure 2 shows the case fatality rate by age, and table 1 shows the associations with death among individuals with Ebola virus disease. The case fatality rate was highest in children under 2 years old and older adults, and lowest at ages 10 to 14 years. The case fatality rate was higher in larger households, with little difference by sex, and varied by exposure level, occupation group, time period, and position in household.

**Figure f2:**
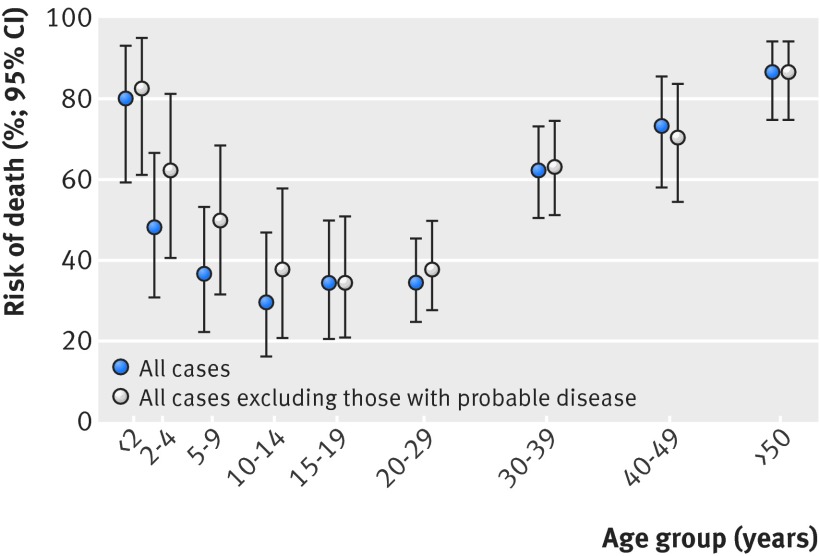
**Fig 2** Case fatality rates by age among people with Ebola virus disease

**Table 1 tbl1:** Univariable associations between individual level and household level factors and mortality among individuals with Ebola virus disease

	No of deaths/cases	Proportion (%)	Odds ratio (95% CI) adjusted for clustering, age, and sex	P
**Age (years)**				
<2	20/25	80.0	7.5 (2.3 to 24.2)	<0.001
2-4	16 /33	48.5	1.9 (0.78 to 4.7)	
5-9	15/41	36.6	1.1 (0.47 to 2.5)	
10-14	11/37	29.7	0.75 (0.30 to 1.9)	
15-19	15/44	34.1	1.0 (0.44 to 2.4)	
20-29	31/90	34.4	1	
30-39	48/77	62.3	3.7 (1.8 to 7.6)	
40-49	33/45	73.3	5.8 (2.4 to 13.8)	
≥50	46/53	86.8	15.9 (5.9 to 42.7)	
**Sex**				
Female	135/263	51.3	1	0.82
Male	103/185	55.7	1.1 (0.67 to 1.7)	
**Primary case**				
Yes	61/97	62.9	1	0.10
No	177/351	50.4	0.58 (0.30 to 1.1)	
**Exposure level†**				
Corpse	39/69	56.5	1	0.009
Fluid	30/80	37.5	0.44 (0.19 to 1.0)	
Direct wet	89/163	54.6	1.7 (0.79 to 3.6)	
Direct dry	37/56	66.1	2.2 (0.89 to 5.5)	
Indirect wet	4/11	36.4	0.80 (0.16 to 3.9)	
Indirect dry	18/24	75.0	2.1 (0.58 to 7.4)	
Minimal/none	21/40	52.5	1.2 (0.45 to 3.3)	
**Month of illness**				
November	43/63	68.3	1	0.11
December	152/297	51.2	0.45 (0.19 to 1.1)	
January	31/55	56.4	0.67 (0.23 to 2.0)	
February/March	12/33	36.4	0.24 (0.067 to 0.83)	
**Position in household**				
Head	34/56	60.7	1	0.03
Member	204/392	52.0	2.4 (1.1 to 5.6)	
**Occupation**				
Manual	91/168	54.2	1	0.005*
Non-manual	34/44	77.3	4.3 (1.6 to 11.6)	
Healthcare worker	18/22	81.8	4.2 (1.0 to 17.9)	
Child/student	89/202	44.1	1.9 (0.76 to 4.8)	
Unknown	6/12	50.0	0.52 (0.096 to 2.8)	
**Household size (no of people)**				
1-5	6/23	26.1	1	<0.001
6-10	68/163	41.7	1.9 (0.62 to 5.7)	
11-15	66/129	51.2	2.6 (0.87 to 8.0)	
≥16	98/133	73.7	7.4 (2.4 to 23.1)	
**Living conditions‡**				
Low	29/67	43.3	1	0.56
Medium	14/251	57.4	1.6 (0.69 to 3.5)	
High	64/127	50.4	1.3 (0.56 to 3.2)	
**Area of residence**				
Rural	54/97	55.7	1	0.60
Urban	183/348	52.6	0.83 (0.41 to 1.7)	

*Excluding unknown category.

†Corpse=direct contact with body of a person who died of Ebola virus disease; fluid=direct contact with body fluids of patient with wet symptoms; direct wet=direct contact with patient with wet symptoms; direct dry=direct contact with patient with dry symptoms; indirect wet=indirect contact with patient with wet symptoms; indirect dry=indirect contact with patient with dry symptoms; minimal/none=minimal or no known contact.

‡Household living conditions based on measures of crowding and sanitation (access to water, soap, and latrine). Possible scores were 0-10. More than half the population had scores of 6 or 7. Low was taken as <6, medium 6-7, high >7.

In the full multivariable analysis, only age, household size, date, occupation, and exposure level were associated with death (table 2). Results were similar in a sensitivity analysis after excluding probable disease (table 2). Despite variation in the outcome by exposure level, there was no consistent trend with increasing exposure. For comparison, figure 3 shows the association of exposure level with risk of Ebola virus disease among household contacts (excluding primary cases) in these households.

**Table 2 tbl2:** Multivariable analysis of association between individual and household level factors and mortality among individuals with Ebola virus disease, overall and after excluding probable disease

	All cases			All cases excluding probable disease	
	Odds ratio (95%CI)*	P		Odds ratio (95%CI)*	P
**Age (years)**					
<2	10.2 (2.5 to 41.0)	<0.001		8.0 (1.7 to 37.3)	<0.001
2-4	1.3 (0.42 to 3.8)			1.4 (0.41 to 4.9)	
5-9	0.92 (0.31 to 2.8)			1.2 (0.39 to 4.0)	
10-14	0.70 (0.22 to 2.2)			0.74 (0.23 to 2.4)	
15-19	1.1 (0.40 to 3.0)			0.87 (0.30 to 2.5)	
20-29	1			1	
30-39	4.1 (1.9 to 9.0)			3.7 (1.6 to 8.1)	
40-49	6.1 (2.4 to 15.6)			5.1 (2.0 to 13.5)	
≥50	10.1 (3.6 to 28.4)			8.4 (3.0 to 23.6)	
**Sex**					
Female	1	0.80		1	0.44
Male	1.1 (0.67 to 1.7)			1.2 (0.74 to 2.0)	
**Exposure level†**					
Corpse	1	0.01		1	0.01
Fluid	0.38 (0.16 to 0.89)			0.46 (0.19 to 1.1)	
Direct wet	1.1 (0.52 to 2.4)			1.3 (0.60 to 2.6)	
Direct dry	2.1 (0.83 to 5.0)			2.7 (1.1 to 7.0)	
Indirect wet	0.48 (0.096 to 2.4)			0.52 (0.10 to 2.6)	
Indirect dry	1.2 (0.33 to 4.2)			2.1 (0.44 to 9.9)	
Minimal/none	1.2 (0.44 to 3.2)			1.2 (0.43 to 3.3)	
**Month of illness**					
November	1	0.04		1	0.01
December	0.65 (0.30 to 1.4)			0.48 (0.21 to 1.1)	
January	1.2 (0.44 to 3.3)			0.69 (0.23 to 2.0)	
February/March	0.21 (0.064 to 0.72)			0.14 (0.041 to 0.49)	
**Occupation**					
Manual	1	0.04‡		1	0.07‡
Non-manual	2.7 (1.0 to 7.2)			2.8 (1.1 to 7.5)	
Child/student	1.4 (0.55 to 3.5)			1.6 (0.64 to 4.2)	
Healthcare worker	5.2 (1.1 to 25.2)			3.8 (0.79 to 18.4)	
Unknown	1.4 (0.20 to 10.4)			0.59 (0.070 to 5.1)	
**Household size (no of people)**					
1-5	1	0.003		1	<0.001
6-10	2.1 (0.63 to 7.1)			2.4 (0.71 to 7.9)	
11-15	3.1 (0.93 to 10.4)			3.6 (1.1 to 12.2)	
≥16	7.0 (2.0 to 24.5)			8.4 (2.4 to 29.2)	

*Odds ratios adjusted for all other factors in the table.

†Corpse=direct contact with body of a person who died of Ebola virus disease; fluid=direct contact with body fluids of patient with wet symptoms; direct wet=direct contact with patient with wet symptoms; direct dry=direct contact with patient with dry symptoms; indirect wet=indirect contact with patient with wet symptoms; indirect dry=indirect contact with patient with dry symptoms; minimal/none=minimal or no known contact.

‡Excluding unknown category.

**Figure f3:**
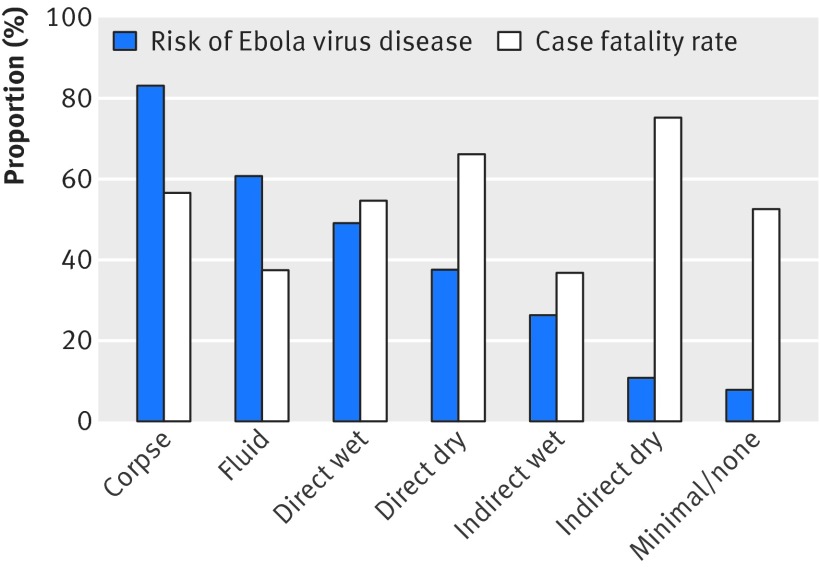
**Fig 3** Relation of exposure level with risk of Ebola virus disease and with case fatality rate. To assess risk of disease by exposure level, primary cases in each household were excluded. Probable Ebola virus disease and deaths are included. Corpse=direct contact with body of a person who died of Ebola virus disease; fluid=direct contact with body fluids of patient with wet symptoms; direct wet=direct contact with patient with wet symptoms; direct dry=direct contact with patient with dry symptoms; indirect wet=indirect contact with patient with wet symptoms; indirect dry=indirect contact with patient with dry symptoms; minimal/none=minimal or no known contact

## Discussion

### Principal findings

In this study, we identified four survivors of Ebola virus disease who died after discharge. All four late deaths could have been caused by Ebola virus disease and its sequelae, although only one patient had a positive PCR result in the postmortem swab. Patient A might have had pancreatitis as a direct effect of the Ebola virus disease.^20^ Patient B appears to have had a stroke.^21^ Patient C could have had an unrelated chest infection, perhaps tuberculosis, although the duration was short. Patient D could have died of tuberculosis and with, rather than of, Ebola virus disease. If all these deaths were due to Ebola virus disease, this would give a risk of late death of 2.6% (four of 151), but only patient C could be considered a recrudescence because only he had a period of full recovery and so fulfilled the case definition. However, patient C had a negative PCR result postmortem. Bearing in mind the limitations of assigning cause of death by verbal autopsy, particularly with non-medical informants, this would give a maximum estimate of 0.7% recrudescence within a mean follow-up of 10 months.

Among the individuals with Ebola virus disease in this study, we found a U shaped pattern of death by age with a high case fatality rate in the youngest and oldest age groups. We found no association with household level socioeconomic factors other than number of people in the household. The date of Ebola virus infection in the household strongly correlated with mortality. Earlier cases of the disease occurred at the height of the epidemic in Sierra Leone when services were most stretched. By mid-January, case numbers had fallen considerably,^4^ treatment centre beds had increased, and staff members were more experienced. The variation by occupation group might reflect the benefits of prompter action, if some groups were more reluctant to seek admission. The non-manual group included 10 religious leaders and chiefs, who all died. Place of residence had no effect on mortality, but was not a good proxy for access to treatment centres because availability of places at different centres varied over time, and household members were often sent to different treatment centres.

We found no evidence of a consistent association between case fatality and the extent of exposure to body fluids. Given the strong correlation between these measured exposure levels and risk of Ebola virus disease (fig 3), our predefined exposure scale seemed to be a reasonable measure of infecting dose.

### Strengths and limitations of the study

This large study had a complete follow-up at six to 13 months after discharge, so late deaths will not have been missed. The case fatality rate in this study underestimated the overall case fatality rate, because our starting point was survivor households (because they could be approached through the treatment centre outreach team). Excluding one index survivor per household would give a case fatality rate of 75% (227/301) for reported Ebola virus disease, or a rate of 67% (238/354) including probable disease. 

We did not include households in which all individuals with Ebola virus disease died; therefore, small households could have been under-represented in our sample. This exclusion might partly explain the association found between case fatality rate and household size, but it is also possible that large households with many affected members found it particularly difficult to provide care. 

Associations between the other risk factors and death should not be biased. We were able to include probable cases and deaths that might have been missed from notification data, and assess their influence on the results. In our study, inclusion of probable disease lowered the case fatality rate, but had little effect on the associations with mortality. We did not know which of the deaths occurred in treatment centres, so could not assess the benefit of admission directly.

### Comparison with other studies

To our knowledge, this is the first cohort study of Ebola virus disease with active follow-up for late deaths, and the first large community based study to look at risk factors for death from the disease. Previous studies of sequelae have reported on patients seen in clinics so would have missed any deaths.^11^ ^13^

The U shaped pattern of death by age was similar to that seen in the WHO notification data^4^ and, as in these notification data,^4^ we found a marginally higher case fatality rate in males. The lack of association between socioeconomic status and case fatality suggests that although socioeconomic status has been associated with the risk of individuals having Ebola virus disease,^22^ once ill, living conditions had little effect on the outcome. The variation in case fatality rate by occupation group could reflect different responses to illness; delays in coming forward for treatment by healthcare workers have been reported previously.^23^

This study also looks at the association between exposure level (as a proxy of dose) and case fatality rate in Ebola virus disease. A lack of association between infecting dose and severity of disease suggests that symptomatic illness can be established by one or very few organisms.^24^ Dose can therefore affect the probability of contracting disease without influencing severity and risk of death once a person becomes ill. This is consistent with animal challenge studies which find that animals receiving low doses of Ebola virus either died or remained asymptomatic,^25^ ^26^ although higher doses were associated with a shorter time to death.^27^ It is also compatible with the association between Ebola viral load on admission to treatment centres and outcome, since a high viral load at this stage suggests a failure to control viral multiplication rather than a high initial infecting dose. However, deep sequencing of viruses has found the same minority variants in different patients, suggesting that the transmission bottleneck allows through more than one virus.^28^ ^29^ Whatever the mechanism, it appears that once a person becomes ill, factors other than infectious dose determine the outcome, and different immune responses have been noted in survivors and fatalities from early on in the disease.^30^

### Conclusions and policy implications

The age pattern of the case fatality rate suggests that differences in susceptibility are important in determining the outcome of Ebola virus disease. However, the associations with time period, occupation, and household size suggest that care given was crucial in reducing mortality, emphasising the importance of Ebola treatment centres. Infecting dose of the virus did not appear to have a role. All deaths after discharge occurred within a few weeks, and we have follow-up information six to 13 months later on all survivors. Recrudescence of severe active disease leading to death appears to be rare, which should be reassuring for Ebola survivors and their contacts, but does not remove the need for continued monitoring of survivors’ health.

**What is already known on this topic**

Understanding who dies from Ebola virus disease, and when, is crucial for determining the effect of interventions and planning the public health response
Case fatality rates vary by age and viral load on admission to treatment centres, but it is not known if they vary by infecting dose
Frequency of recrudescence and late deaths from Ebola virus disease after discharge from treatment centres is unknown

**What this study adds**

This is the first cohort study of Ebola virus disease with active follow-up for late deaths, and the first large community based study to investigate risk factors for death from the disease
Recrudescence of severe Ebola virus disease appears to be rare up to 10 months after discharge
Infecting dose, as measured by extent of exposure to body fluids, strongly correlated with risk of developing the disease, but there was no consistent trend with case fatality rate

## Notes

We thank all the participants for the time and thought they gave to the study, the field team for their dedicated work, the Save the Children country office for their support; and the Ministries of Health and Sanitation and of Social Welfare, Gender and Children’s Affairs for their permission to carry out the study and support throughout.

Contributors: JRG, HB, and FC designed the study with contributions from all the other authors. HB, SJ, and ES led the fieldwork with MSB, OK, and CT. SO maintains the survivor database. JRG and HB did the analysis. JRG led the writing with contributions from all the other authors. All authors have approved the final manuscript. JRG is the study guarantor, had full access to all the data, and is responsible for the decision to submit for publication.

Ethical approval: Permission for the study was granted by the Sierra Leone ethics and scientific review committee and the ethics committee of the London School of Hygiene & Tropical Medicine. All living participating household members gave individual, written informed consent (parents or guardians gave consent for those aged under 18 years).

Data sharing: A full dataset will be available with restrictions to ensure confidentiality and prevent deductive disclosure in the London School of Hygiene and Tropical Medicine’s data repository, DataCompass. The consent mentioned publication but did not explicitly mention data sharing; the presented data are anonymised and risk of identification is low.

The guarantor affirms that the manuscript is an honest, accurate, and transparent account of the study being reported; that no important aspects of the study have been omitted; and that any discrepancies from the study as planned have been explained.
